# Assessment of Effective Coverage of Antenatal Care and Associated Factors in Squatter Settlements of Islamabad Capital Territory, Pakistan: An Analytical Cross-Sectional Study

**DOI:** 10.7759/cureus.28454

**Published:** 2022-08-26

**Authors:** Ammarah Khan, Saima Hamid, Tahira Ezra Reza, Kauser Hanif, Faran Emmanuel

**Affiliations:** 1 Public Health, Center for Global Public Health, Islamabad, PAK; 2 Reproductive, Maternal, Newborn, Child Health and Adolescents Health, Fatima Jinnah Women University, Rawalpindi, PAK; 3 Epidemiology and Public Health, Center for Global Public Health, Islamabad, PAK; 4 Obstetrics and Gynecology, Center for Global Public Health, Islamabad, PAK; 5 Epidemiology and Public Health, University of Manitoba, Winnipeg, CAN

**Keywords:** pakistan, squatter settlements, factors associated, effective coverage, ante-natal care

## Abstract

Background: Effective coverage of antenatal care (ANC) goes beyond contact coverage and assesses the quality of service provided. We used World Health Organization’s recommended positive pregnancy guidelines to assess effective coverage and factors associated with the utilization of ANC among women in squatter settlements of Islamabad Capital Territory.

Methods:We conducted a household survey in the study area with 416 women who had given birth in the past one year. Face-to-face interviews were conducted after the selection of study subjects was done through a systematic random sampling approach. Statistical analysis was carried out using Statistical Package for the Social Sciences 22 (SPSS 22; IBM corp. Armonk, NY). Effective ANC coverage was defined as four or more ANC visits along with all WHO-recommended interventions received at least once during ANC. Adjusted odds ratios (adjOR) with 95% CI were calculated using binary logistic regression to determine the independent effects of all associated factors on the outcome.

Results: Of the 416 women interviewed, 399 (95.6%) had availed ANC services at least once. The coverage of 4+ ANC visits was 92% but effective coverage was only received by 35% women. The proportion of women who received nutritional interventions, maternal and fetal assessment and other preventive measures was 68%, 51% and 80.8% respectively. Maternal education (adjOR, 95% CI = 4.8[2.4-9.3]), family income (2.3[1.1-5.1]), multiparity (1.7[1.1-2.9]), place of first ANC visit (4.2[1.7-10.5]) and distance from a health facility (2.2[1.3-3.6]) were independently associated with the non-utilization of effective ANC.

Conclusion: Despite a very high crude coverage of ANC services, the study shows a very low proportion of women receiving effective coverage. This stresses the importance of measuring the proportion of the population that receives health services with quality to monitor progress toward achieving universal health coverage.

## Introduction

Antenatal care (ANC) is among the core intervention in the operational continuum of care for the improvement of maternal health [1]. The antenatal period provides an opportunity to reach out to pregnant women, and provide them with services that would ensure a safe pregnancy and delivery, and are vital to the health of the child [2]. The ANC uptake is an important determinant of maternal health status and non-utilization of ANC during pregnancy has shown a significant association with maternal mortality in the developing world [2]. Available evidence suggests that two-thirds of the world’s maternal and neonatal morbidities can be alleviated if these interventions are optimally implemented and utilized [3].

While the benefits of ANC are documented extensively, the exact number of recommended visits needed, and the components of the ANC package have been debated [4]. Globally, the number of ANC visits is being used as an indicator for monitoring the maternal health program performance [5]. While the number of ANC visits is important, the number of visits alone does not guarantee the receipt of interventions that are effective in improving maternal health [6]. Therefore, other indicators measuring the quality of services received should also be considered [7]. More recently, WHO has recommended a positive pregnancy experience which means, that women maintain a healthy pregnancy have sociocultural and physical normality and an effective transition from positive pregnancy to a positive birth, and finally attain positive motherhood. [8]. It recommends that in addition to pregnant women utilizing four or more ANC visits there is a need to focus more on what services are provided and its quality. Thus “effective coverage” of ANC services includes the number of ANC visits as well as the quality of care in compliance with international guidelines [9].

Serial data generated from Pakistan Demographic Health Survey (PDHS) [10] in Pakistan shows significant increase in ANC coverage measured by calculating the proportion of women who received at least one ANC visit from a skilled provider during pregnancy; an increase from 26% in 1990-1991 to 86% in 2016-2017 was observed. Marked disparities due to urban-rural, education and income status were also reported. [10]. Although more than half of the women reportedly have had four or more visits, the maternal mortality in the country still stands at 186/100,000 live births [11]. A possible explanation for this high maternal mortality despite a high ANC contact coverage could be a lack of quality and the incomplete range of services received, which has not been fully investigated. Moreover, while urban and rural areas are focused on the squatter settlements known as “Katchi abadi” that exist in nearly all urban centers in Pakistan have been consistently ignored in all National and subnational surveys. The uniqueness of these populations in terms of socio-economic status, socio-cultural dynamics and accessibility to health services make them dissimilar to urban populations. We, therefore, conducted this study to measure ANC coverage in the squatter settlements of Islamabad Capital Territory (ICT), assess effective coverage and understand the factors associated with the utilization of ANC services in these settlements.

## Materials and methods

Methods

A cross-sectional study was conducted in the squatter settlements of ICT between April 2019 to August 2019. ICT has a total number of 37 squatter settlements defined as ‘a low residential area, which has developed without the legal right to the land or permission from the concerned authorities to build, and as a result, of their illegal status, infrastructure and services are usually inadequate [8]. Of the total 37 settlements, we removed the seven Afghan settlements, owing to their high mobility and security concerns. We then selected nine of 30 slums through a systemic random selection technique. After the selection of the settlements, we then calculated the sample size proportionate to the population within each settlement. Of the one million people residing in Islamabad 379,640 (38%) live in squatter settlements/underserved areas [12]. The study population included resident women of squatter settlements within the reproductive age group of 15 to 49 years, who had a live birth in the last one year. Women who were not Pakistani nationals and permanent residents of the study area were not included.

Sampling procedure and sample size

The sample size was calculated to estimate the proportion of women who received effective ANC coverage as well as to identify the associated factors. The sample size to estimate the proportion of women who received effective ANC coverage was calculated at a 95% confidence interval, with a 5% bound on error of estimation and an assumed prevalence of ANC care at 80% in ICT. We further calculated our sample to identify all factors associated with effective ANC with an odds ratio of at least 2, with 80% power and an alpha of 5%. We calculated sample sizes for all different exposures among ANC non-users (25% to 60%) which ranged between 308 and 375. The larger sample size was selected which was further inflated by 10% for non-response and data errors. Our final sample size required 412 women to be included in the study. A random sampling technique was used to recruit study participants. In the first stage, of the 37 squatter settlements, we randomly selected nine (after removing seven Afghan settlements due to high mobility and security concerns) and proportionally allocated the sample based on size. The lady health supervisors (LHS) and lady health workers (LHWs) from the ICT health program, provided the study team with a list of households with eligible women who had a live birth in the last one year. In the next stage, households were selected using simple random sampling. Within households with more than one eligible respondent, a study participant was selected randomly.

Data collection and management

Data were collected from eligible mothers using the pre-tested structured questionnaire to acquire information on socio-demographic characteristics, obstetric history and ANC. The study questionnaire was adapted and constructed from a validated and reliable PDHS questionnaire [10]. The questionnaire was developed in English, translated into Urdu and back-translated to eliminate translation errors. The questionnaire was pre-tested to check for the appropriateness of questions, comprehensiveness of the questionnaire, content and field procedures of data collection. A pretest was conducted at 5% (20 samples) of the sample size. The tool was tested in a squatter settlement, Faisal colony, G-7/1. The issues in the questionnaires faced in the pretest were modified prior to moving into the field for data collection. The phrasing, translation and response noting were modified accordingly. The questionnaire was translated to Urdu and the data collected in the field was in Urdu. This data was later translated into English. The research team consisted of four interviewers who were public health graduates and one field supervisor. A three-day training workshop was conducted by the Principal Investigator to ensure proper training of the team on field implementation and data collection. A field office was established at Health Services Academy, Islamabad, where the field teams gathered each day to discuss daily progress and highlighted any field issues. LHWs provided facilitation in the field by identifying eligible households, introducing the research teams into the household, and in the recruitment process. After introducing the study and taking informed consent, face-to-face interviews were carried out with the respondents in a separate room, to maintain confidentiality, until or unless the respondents themselves allows someone to enter the area. Consent was read out and on behalf of the woman, interviewer was a sign to the consent. The interview would begin only after obtaining consent from the respondent. The questionnaires were collected from the field at the end of each day. The questionnaires were collected by the research team from the field daily. They were transported in official vehicles provided to the research team to the field office. All the filled questionnaires were edited and handed over to the data manager, who would then enter them using CSpro data entry software on a daily basis. Data security was ensured by keeping the filled questionnaires in a secure place at the research office. All the questionnaires were coded and no respondent name or identifying detail was mentioned to ensure the confidentiality of the data.

Study variables

Other than the socio-demographic information and maternal history obtained for each individual, the questionnaire included all three WHO recommended intervention domains, i.e., i) nutritional interventions, ii) maternal and fetal assessment and iii) preventive measures [1]. Information on the number of ANC visits was also obtained. Nutritional interventions included counseling on health eating during pregnancy, daily oral iron and folic acid supplementation, and the use of calcium supplements. The maternal and fetal assessment included measurement of maternal weight and height, screening for blood group, hypertension, gestational diabetes, anemia, examination of the abdomen and fetal heartbeat as well as antenatal ultrasound and detailed urine analysis. Preventive measures comprised at least two doses of tetanus toxoid vaccination. All of these variables were dichotomized to a “yes” or “no” response. A positive coverage within each domain was considered if a woman received all services within the domain at least once. The final outcome variable, i.e., utilization of effective ANC coverage was defined as four or more ANC visits along with receiving all WHO recommended interventions within each of the three domains, at least once during ANC. The outcome variable, effective coverage of ANC is defined as having four or more ANC visits along with receiving all the WHO recommended interventions within each domain at least once during ANC.

Data analysis

The data management team comprised a data manager and two data entry operators that worked along with the field team. The questionnaires received from the field were cleaned, coded and edited prior to entry in the Census and Survey Processing System (CSPro) database that was specifically designed for the study. The final data set was analyzed using Statistical Package for Social Sciences (SPSS v.20; IBM Corp., Armonk, NY). Our analysis was directed to initially calculate the proportion of women who received effective ANC, followed up by comparing ANC receivers and non-receivers to understand the associated factors. Following a descriptive analysis of all variables of concern, a candidate variable was selected to calculate the level of significance for each independent variable against the outcome variable measuring unadjusted odds ratios and its associated 95% confidence interval. Multivariate analysis was done using a binary logistic regression model to see the independent effect of each factor associated with the non-provision of effective ANC coverage.

## Results

Data were collected from 416 women with a non-response rate of less than 1%. Of all the women interviewed 93.8% (n=390) were currently married with an average age of 26.7 years (standard deviation = 4.8). Nearly one-quarter of the respondents had no formal education, and another quarter had more than 10 years of education. The majority of the women live in a joint family system, and nearly one-third were employed. The average monthly household income was PKR. 21,183± 10,180 (median income = 20,000). Nearly one-third of the sampled women were primiparous and more than half were multiparous with less than four children. A fairly high proportion of women, i.e., 28% reported a history of a miscarriage or abortion previously, while 12.3% reported of a stillbirth (Table 1).

**Table 1:** Socio-demographic characteristics and Maternal history of study participants in squatter settlements of ICT.

Sociodemographic Characteristics	N(%)
Marital Status	
Currently Married	390 (93.8)
Separated/Divorced/Widowed	26 (6.2)
Mother’s Current Age* Average ±SD = 26.7±4.8	
Less than 30 years	291 (70.0)
30 years and more	125 (30.0)
Mother’s Age at Marriage	
20 years and less	191 (45.9)
More than 20 years	225 (54.1)
Mother’s Education	
No Formal Schooling	102 (24.5)
Less than secondary school (10 years)	211 (50.7)
Secondary School and higher (10 years and more)	103 (24.8)
Mothers Employment status	
Employed	136(32.7)
Unemployed	280(67.3)
Family system	
Joint Family	286(68.8)
Nuclear Family	130(31.3)
Husband’s Education	
No Formal Schooling	83 (20.0)
Less than secondary school (10 years)	135 (32.5)
Secondary School and higher (10 years and more)	198 (47.6)
Husband’s Employment status	
Employed	387 (93.0)
Unemployed	29 (7.0)
Monthly Household Income	
Average income (Median) in PKR**	21,183 ± 10,180 (20,000)
Obstetric history	n (%)
Miscarriages/Abortions	119(28.0)
Still Births	51(12.3)
Parity	
Primiparous	141(33.9)
Multiparous	275(66.1)

Every study participant received at least one ANC visit, with an average number of 8.9 ± 3.1 ANC visits. Nearly one-third of women received ANC at a government tertiary care hospital (67%) and 88% of those were attended by a medical doctor.

Table 2 provides the coverage of WHO-recommended interventions within each domain. Four or more than four ANC visits were recorded in 92% of the study participants, demonstrating a very high crude coverage. Within the domain of nutritional intervention, 68% of the women received all three recommended nutrition interventions, i.e., received information on the importance of maternal nutrition (75.5%), received iron/folic acid supplement (88.5%) and calcium supplements (87.3%). Nearly half of the women (51.4%) received all nine maternal and fetal interventions. Among these the most commonly received interventions were screening for hypertension and abdominal USG (94%) while height (51.4%) and screening for gestational diabetes (58.2%) were the least assessed. Effective coverage for ANC was found to be 35.1% at 95% CI, defined as women who had four or more ANC visits and also received all WHO-recommended interventions within each of the three domains, at least once.

**Table 2:** Measurement of effective coverage of antenatal care Interventions among the study population in squatter settlements of ICT.

Composite Variables	n (%)	Coverage of ANC services n (%)	Effective Coverage of ANC n (%)
Coverage of ANC visits			146(35.1)
4 and more ANC visits at recommended timings	383 (92.1)	383 (92.1)	
Nutrition			
Received information on the importance of maternal nutrition	314(75.5)	283 (68.0)	
Iron/ folic acid supplement	368(88.5)		
Calcium supplements	363(87.3)		
Maternal and Fetal Assessment			
Measurement of height	214(51.4)	214(51.4)	
Screening for hypertension	392(94.2)		
Antenatal ultrasound	391(94.0)		
Abdominal examination	370(88.9)		
Examination of fetal heartbeat	379(91.1)		
Screening for anemia	368(88.5)		
Screening for gestational diabetes mellitus	242(58.2)		
Detailed urine analysis	331(79.6)		
Screening for Rh-type	355(85.3)		
Preventive Measures			
Tetanus Toxoid	336(80.8)	336(80.8)	

Multivariate analysis using binary logistic regression was conducted to study the independent effect of each independent variable on the outcome, i.e., effective coverage of ANC. The results are presented in Table 3 which presents the association of independent variables with the outcome variable by calculating the unadjusted and adjusted Odds ratio with a corresponding 95% confidence interval. Analysis shows that having women with no maternal education, those in lower family income quintiles, multi parity, having received their first ANC care at a primary level facility and living more than 5kms away from the health facility were the factors independently associated with non-utilization of effective ANC coverage in ICT.

**Table 3:** Multivariate analysis of effective coverage of antenatal care among women living in squatter settlements in ICT.

Sr. No.	Independent Variables	Did not receive Effective Coverage n(%)	Received Effective Coverage n(%)	Unadjusted OR (95%CI)	Adjusted OR (95% CI)
1	Maternal Education				
	No formal education	88 (32.6)	14 (9.6)	4.55 (2.48 to 8.36)	4.77 (2.44 to 9.34)
	Received formal education	182 (67.4)	132 (90.4)		
2	Family Income				
	1^st^ Quintile	78 (28.9)	30 (20.5)	2.4 (1.22 to 4.76)	2.36 (1.09 to 5.06)
	2^nd^ Quintile	48 (17.8)	28 (19.2)	1.59 (0.78 to 3.23)	2.05 (0.91 to4.58)
	3^rd^ Quintile	58 (21.5)	27 (18.5)	1.99 (0.98 to 4.02)	2.06 (0.93 to 4.57)
	4^th^ Quintile	58 (21.5)	35 (20.4)	1.53 (0.78 to 3.03)	1.27 (0.59 to 2.72)
	5^th^ Quintile	28 (10.4)	26 (17.8)		
3	Parity				
	Primiparous	81 (30.0)	60 (41.1)	1.68 (1.07 to 2.47)	1.75(1.06 to 2.88)
	Multiparous	189 (70.0)	86 (58.9)		
4	Distance from health facility				
	5km and less	114 (47.5)	48 (35.6)	1.64 (1.06 to 2.53)	2.21(1.35 to 3.63)
	More than 5km	126 (52.5)	87 (64.4)		
5	Place of first ANC				
	Primary level facility	39 (15.4)	7 (4.8)	3.61 (1.57 to 8.31)	4.25 (1.72 to 10.50)
	Secondary level and above	214 (84.6)	139 (95.2)		

## Discussion

Our study has looked into the effective coverage of ANC using WHO’s positive pregnancy guidelines and also identified its associated factors. To our knowledge, this is the first study conducted in the squatter settlements of ICT, which has neglected geography in most National and sub-national surveys.

The results of our study show that nearly 35% of the women received effective coverage of ANC services. While 80% ANC contact coverage rates for ICT (which includes both urban and rural strata) are reported by the recent PDHS survey [10] our study showed that 96% women in the squatter settlements, availed ANC at least once during their pregnancy, and overall an 88% had four or more visits. While PDHS only looks at contact coverage, we followed the recommendations of the “Effective Coverage Think Tank Group” to evaluate health-service coverage which allowed us to do a population-level assessment of ANC utilization services [13]. We moved one step ahead and measured the proportion of women who came into contact with an ANC provider and received services according to the quality-of-care standard [14]. Our findings are coherent with data analyzed from district health information systems (DHIS) from 41 countries which shows large coverage gaps in the number of ANC visits and quality of service provision [15-17]. If we only consider contact coverage, both PDHS (2017-2018) and this study reports very high utilization rates of above 80%; however, it does not provide any information regarding which services were received, and what was the quality of those services. Thus, we identify fairly large gaps between contact coverage and effective coverage which reiterates the fact that counting the number of ANC visits is not enough, and there is a need to measure the quality of services received during those visits.

We found that of all the services provided, 94.2% of the women had been screened for hypertension and 94% had an antenatal ultrasound done. This can be attributed to the fact that the majority of the women received ANC at the secondary level or higher facility and our study reports that receiving ANC at the primary level increases the risk of not receiving effective coverage due to the unavailability of diagnostic laboratories and ultrasound facilities. The majority of the women, 80%, had received two doses of tetanus toxoid vaccination which is critical in preventing neonatal tetanus and improving neonatal outcomes [15,18,19]. These findings are coherent with similar studies [9,15].

Poor maternal education, belonging to a lower income class, parity, first ANC provided at a PHC facility and distance from a health facility were factors independently associated with the poor uptake of quality ANC. Our finding that maternal education is strongly associated with the uptake of effective coverage of ANC services, is consistent with a number of studies from South Asia where women with higher levels of education had received better quality of ANC services [20-22].

Education fosters positive attitudes and creates awareness towards the uptake of modern healthcare and inclines women towards receiving skilled care and improves comprehension of health education and information. [20,21]. A higher economic status as compared to women in the lowest income quintile was found to be highly associated with ANC coverage, which corroborates with previous literature [20,23]. A review of data from LMICs including South East Asia presents a similar picture where women from the highest wealth quintile have higher access to health care, which leads to higher utilization of ANC services as seen in the current analysis [24,25]. High socioeconomic status improves affordability and better access to health care and health information compared to the women in lower socioeconomic strata.

Primary healthcare facility as the place of first ANC visit posed a higher risk of not receiving effective ANC. A possible explanation for this association could be the lack of diagnostic facilities such as an ultrasound machine and laboratory facility for testing blood and urine samples. This finding was corroborated by similar studies which conclude that local facilities often lack diagnostic services which results in poor quality of ANC provision [26]. To lessen the burden on tertiary health facilities, it is important to equip primary facilities with diagnostic services, trained health providers and technicians to ensure effective ANC provision at all levels of care. Our study also reports that multiparous women have decreased odds of receiving quality ANC services compared to nulliparous mothers. There could be a number of reasons for this association. Since the first pregnancy is a new experience for nulliparous women, they usually are more careful of the first pregnancy compared to the second or later pregnancies [27-29]. Multiparous women are also burdened with responsibilities and looking after other children and find less time and motivation to avail of ANC [28-30]. Women’s previous experience of uncomplicated pregnancies also influences their perception of the importance and need to avail ANC. In univariate analysis, we found complications significantly associated with effective coverage of ANC; however, we did not find an independent association possibly because of a small number of women who faced a previous pregnancy-related complication.

Women living within 5km of a health facility were more likely to receive effective coverage compared to those living at more than 5km distance. Similar studies showed that women living closer to the health facility were more likely to take up ANC and receive quality coverage of ANC [23,28,30]. Lack of proper transport, the expense incurred for traveling to health facilities and the distance of health facilities discourage the uptake of ANC at health facilities and could be a barrier to effective coverage of ANC [27,29].

Strengths and limitations

The strength of this study is a short recall period compared with the PDHS survey, which includes women having given birth in five years preceding the survey, our study included women having given birth in the past one year, and asked questions about their most recent pregnancy. This lowers the recall bias which is inherent in population-based surveys. A few limitations of the study need to be discussed. This study was conducted in the squatter settlements of ICT; thus, the findings are from a marginalized population and might not be applicable to urban or other rural settings in the country. Finally, the study was based on self-reported data, some of the respondents might not have known what services were provided to them, which might lead to a dilution of the results of this study.

## Conclusions

The study illustrates unsatisfactory compliance with WHO-recommended core ANC interventions with only one-third of the women receiving effective coverage despite a very high crude coverage. It might suggest that, while the health system is able to provide access to an intervention, the quality of the intervention is not up to standard and the perceived benefits are not achieved. The result of this study also explains why despite a trending improvement in the coverage of ANC in Pakistan the morbidity and mortality indicators have not shown much improvement. This stresses the importance of measuring the proportion of the population that receives health services with quality to monitor progress toward achieving universal health coverage. This study should be replicated in the national household survey to assess the quality of ANC services and similar indicators to determine the effective coverage of MNCH services and formulate policies at the national level.
